# Managing the “Obscene M.D.”: Medical Publishing, the Medical Profession, and the Changing Definition of Obscenity in Mid-Victorian England

**DOI:** 10.1353/bhm.2017.0079

**Published:** 2017

**Authors:** Sarah Bull

**Keywords:** Victorian, publishing, quackery, professionalism, respectability, pornography, Hicklin, obscene, book trade, sexuality

## Abstract

This article examines links between mid-Victorian opposition to commerce in popular works on sexual health and the introduction of a legal test of obscenity, in the 1868 trial *R. v. Hicklin*, that opened the public distribution of any work that contained sexual information to prosecution. The article demonstrates how both campaigning medical journals’ crusades against “obscene quackery” and judicial and anti-vice groups who aimed to protect public morals responded to unruly trade in medical print by linking popular medical works with public corruption. When this link was codified, it became a double-edged sword for medical authorities. The *Hicklin* test provided these authorities with a blunt tool for disciplining professional medical behavior. However, it also radically narrowed the parameters through which even the most established practitioners could communicate medical information without risking censure.

In the early 1850s, the British College of Health issued a series of four lithographs that claimed to depict the “Morality of Modern Medicine-Mongers.”^1^ Three show medical practitioners exploiting their positions [Other P-713]to “impose . . . upon” female patients.^2^ The fourth (Figure 1), titled “The Obscene M.D.,” illustrates a different kind of professional misbehavior. A publisher, Mr. Quarto, sits in a bookshop, surrounded with such dubiously titled works as *Mysteries of Matrimony*, *The Silent Friend*, and *Manly Vigour*. “Your book goes off famously, Doctor,” Quarto informs an author, “Nothing like a highly-seasoned work, [*sic*] to sell. . . . We can push the thing, because it is written by an M.D.; the police authorities cannot touch us, we are *beyond* all *law*; because we are privileged by the *law* to write obscene books, and call it *science*.” Quarto’s author is enthusiastic. “Capital, by Jove!” he exclaims, “we may defy the police and all the anti-Vice societies: let them touch an M.D. if they can. . . . [M.D.s are] licensed to write, publish and sell all the obscenities we can collect!” As with many of the college’s promotional materials, this lithograph aimed to elevate the holistic system of medicine of its founder, James Morison, by casting other practitioners—both “regular” and “irregular”—as immoral profiteers.^3^ Its efficacy derived from its strategic engagement with questions about medical print that preoccupied Victorians working within and outside the medical profession. In a period in which no clear medical orthodoxy existed, what constituted legitimate medical writing? Should—and could—trade in medical works be regulated? And what would the results of regulation be?

This essay examines the links between mid-Victorian opposition to commerce in popular works on sexual health and the introduction of a test of obscenity that would be used to police the distribution of medical knowledge well into the twentieth century. Formulated by the Lord Chief Justice Alexander Cockburn in his ruling in the 1868 obscenity trial *R. v. Hicklin*, this test—“whether the tendency of the matter charged is to deprave and corrupt those whose minds are open to such immoral influences, and into whose hands a publication of this sort may fall”—made how sexual information was presented and distributed, and to whom, deciding grounds for judgment in obscenity trials.^4^ Having also deemed the intentions and qualifications of its producers immaterial, this precedent opened the public distribution of any work containing sexual details to prosecution, first in Britain and then in other English-speaking [Other P-714]

**Figure 1:**
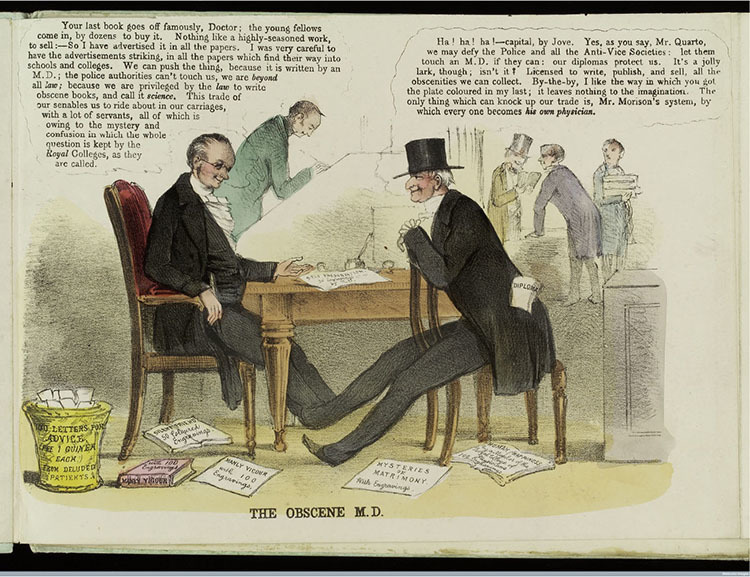
“The Obscene M.D.” Colored lithograph. *Morality of Modern Medicine Mongers*. British College of Health, 1852. Print. Image Courtesy of Wellcome Library, London. Iconographic Collection 563105i.

countries. Scholarship on Victorian debates about “obscene” medical material has largely focused on post-1868 battles over the right to circulate information about contraception. Historians of medicine, sexuality, and censorship have shown how the *Hicklin* test shaped the ways readers encountered such information, by deciding trials that afforded some birth control manuals enormous publicity, by suppressing the distribution of others, and by inspiring the development of new publishing and distribution methods to avoid prosecution.^5^ The test’s use by state authorities and [Other P-715]anti-vice groups as justification for destroying these and other works on sexual health, or limiting their circulation, is usually framed in this literature as opportunistic or incidental. This essay shows that policing medical print was, in fact, one of the goals that underpinned the test’s creation.

Although the *Hicklin* trial surrounded an allegedly obscene religious pamphlet, not a medical one, Cockburn’s ruling responded to calls from medical professionals, anti-vice crusaders and legal experts for measures to limit trade in works on sexual health. The fact that Cockburn’s test simply authorized ideas about obscenity that these groups promoted for years has been overlooked, likely because studies of “obscene” medical material in pre-*Hicklin* Britain tend to concentrate on professional opposition to public anatomical museums.^6^ Scholars have valuably shown how this opposition helped set the parameters of professional medical knowledge and legitimate medical practice at a time when advocates of medical reform appealed for “uniformity” among practitioners and sought to cement their status as trusted social authorities. This essay situates debates about “obscene” medical exhibitions within the context of longer-standing anti-quackery campaigns. It demonstrates how, during a period of rising anxiety about the social effects of cheap print, campaigning medical journals like the *Lancet* attempted to police medical practice by collapsing quackery and obscenity. By labeling certain methods of trade in medical print “obscene quackery,” they attempted to draw boundaries between orthodox medicine and quackery rooted in commercial practices. In doing so, these journals aligned themselves with anti-vice crusaders who aimed to protect the public from immoral literature. These crusaders [Other P-716]themselves exploited how commerce in works on sexual health emphasized problems with existing obscenity laws to push for a legal definition of obscenity, also rooted in questions of presentation and distribution, that suited their own goals.

This history emphasizes just how polymorphous medical practice remained in the nineteenth century, even after the 1858 Medical Act, and how debates about the nature and purpose of medical publications both supported professionalization efforts and impeded medical communication. The medical and the print marketplaces had been enmeshed since at least the early modern period, but their relationship was increasingly complicated in this one.^7^ A disparate range of entrepreneurs published and sold medical works. Many were not medical practitioners, and the authority of those who were was often contested. The styles of commerce in medical print that this essay examines raised difficult questions about what “real” medical publications were, and who should have access to them. However, the debates they inspired were more instrumental than they were philosophical, and fostered long-term problems for communicating medical knowledge. Many medical men eagerly linked commerce in popular works on sexual health with moral corruption because this offered them an opportunity to disassociate themselves from “irregular” practitioners. When the link was codified, it became a double-edged sword: the *Hicklin* test made it easier to police the medical marketplace in some ways, but it also radically narrowed the parameters through which even the most eminent practitioners could communicate without risking censure.

## The Unruly Trade in Medical Print

Medical works have been subject to accusations of obscenity since at least the early modern period, when their increasing availability in vernacular languages aroused anxiety that their intimate descriptions the body would incite immoral behavior among nonprofessionals.^8^ Concern for morally [Other P-717]vulnerable readers—usually conceived as women, youths, and working-class men—officially remained the guiding principle of mid-Victorian opposition to “obscene” medical works, but its targets were broader. In a period in which cheap print abounded, with the emergence of faster production and distribution methods and the gradual abolishment of “taxes on knowledge,” whole systems of trade in works on sexual matters expanded, and aroused opposition.^9^ The most well documented of these trades was undertaken by what I term commercial medical dealers—a group often labeled “quacks” or “irregulars” by historians. The distinctive brand of commerce in which these dealers engaged had been a feature of the medical marketplace since the sixteenth century.^10^ Often working without formal qualifications, they differentiated themselves from other practitioners through their use of sophisticated marketing strategies to build locally, nationally, and even internationally recognized businesses that dealt in a range of medical products and services. Print was a crucial component of these businesses, operating both as a vehicle for publicity and as a commodity itself.

The books and pamphlets that commercial medical dealers self-published were an important source of sexual information for nonprofessional readers. Works like R. and L. Perry & Co.’s *The Silent Friend* (1841), Robert James Culverwell’s *Green Book* (1841), and Horace Goss’s *Woman: Her Physiology and Functions* (1853) offered them discreet and often detailed information about reproductive anatomy and physiology, venereal disease, and sexual debility. Readers could order such works for delivery by post in sealed wrappers, at increasingly competitive prices. In the 1840s, they sold for a median three shillings, putting most within easy reach of middle-class consumers.^11^ By the 1860s, these works were also widely [Other P-718]accessible to working-class readers: median prices dropped below one shilling, and many commercial medical dealers gave their publications away for free. They were cheap because they functioned as advertisements for their authors’ more expensive proprietary medicines and personal consultations, which were also offered in-person and by post. For the same reason, they were easy to find. Commercial medical dealers promoted their works incessantly on the streets, door to door, and within a wide range of newspapers, from conservative London dailies (*The Standard*) to populist penny weeklies (*Reynolds’s Newspaper*) and sporting (*Bell’s Life in London and Sporting Chronicle*), theatrical (*The Era*) and provincial (*Berkshire Chronicle*) papers.^12^ After the advertising duty was abolished in 1853, introducing cheaper rates, their advertisements became even more numerous. Around the same time, commercial medical dealers recognized the publicity that public anatomical museums could afford them, and such museums became yet another outlet through which they distributed publications.^13^

The smaller trade in medical print taken up by Holywell Street publishers, who dealt in various types of sexually detailed works, and are now considered proto-pornographers, also aroused opposition.^14^ Nicknamed for the narrow London lane where they often did business, these dealers’ commerce in works on sexual health has attracted little attention from historians, despite its significance as a source of popular information about contraception. Holywell Street publishers had started trading in medical works in the 1820s, when the publisher John Joseph Stockdale began to edit existing books on sexual health to enhance their prurient appeal, selling them alongside racy fiction and autobiographies.^15^ The [Other P-719]career of William Dugdale (1800–1868), the most notorious publisher to follow in Stockdale’s footsteps, provides a useful focal point for mapping Holywell Street publishers’ trade in medical works into the 1850s. The flamboyant son of a Quaker tailor-bookseller, Dugdale had moved to London at eighteen to work for the radical publisher William Benbow.^16^ Four years later, he formed his own printing, publishing, and bookselling business at 19 Tower Street, Seven Dials, the first of several premises.^17^ His early publications were mainly radical political and literary works; but by 1827, like several publishers in his circle, Dugdale had begun to shift the focus of his business to erotica, capitalizing on a growing market for sexual entertainment.^18^ It quickly became the primary source of English-and French-language erotic fiction in Britain and the United States.^19^ Dugdale’s advertising material reveals, though, that his catalogue was more diverse than this. By the mid-1830s, he and his closest competitors, Edward Dunconbe and Edward Dyer, also dealt in medical works, guides to London’s bawdy houses, erotic images, songbooks, and popular novels.^20^

Dugdale’s medical offerings covered a wider range of topics than did commercial medical dealers: they included manuals on contraception and pregnancy, such as Charles Knowlton’s *Fruits of Philosophy* (1832), Richard Carlile’s *Every Woman’s Book* (1828), and *Aristotle’s Masterpiece* (1684), as well as anti-masturbation tracts (such as English translations of Samuel-August Tissot’s *L’Onanisme* [1760]) and commercial medical dealers’ own works on the reproductive organs, venereal disease, and sexual debility (such as *The Silent Friend*).^21^ Most of these works sold for either one shilling or two shillings sixpence, by post in sealed wrappers or from Dugdale’s bookshops.^22^ The publications on contraception and [Other P-720]venereal disease partly functioned to support a complementary trade in French letters, which Holywell Street publishers often sold from their shops or by mail order.^23^ As a group, however, works on sexual health were valued by these publishers as a ready source of adult entertainment. Like Stockdale before him, Dugdale was clearly apprised of medical eroticism, and sought to capitalize on it. Some of the fiction he sold blurs the boundaries between medical and erotic narrative,^24^ and at least two of his extant medical publications are appended with erotic prints and stories calculated to emphasize their lubric appeal.^25^ It is Dugdale’s advertisements, however, that fully demonstrate the extent to which he and other Holywell Street publishers marketed medical works as titillating reading material, and illustrate how they expected readers would consume these works alongside other sexually themed publications.^26^

Like his competitors, Dugdale’s sales catalogues emphasize the thrilling detail of his medical works’ sexual representations.^27^ Often appended to [Other P-721]the endpapers of his publications, they allude to such works’ “Extraordinary and Curious” nature (Holywell Street publishers routinely used the term “curious” as shorthand for sexually explicit content), the “remarkable,” “unexpurgated” detail of the cases they recount, and the moral danger that the “Secrets of Nature” they unveil might pose to “youth and unmarried females.”^28^ At the same time, these catalogues exploit the suggestive powers of context, blurring generic distinctions between medical works and erotic books and images by listing them side by side. The advertisements that Dugdale and other Holywell Street publishers placed under various names in a variety of newspapers—from the Chartist *Northern Star and National Trades’ Journal* to theatrical (*The Era*), sporting (*Bell’s Life in London*), and satirical (*The Satirist*) periodicals*—*rely on the same strategy. Those published under Dugdale’s name often list medical works for sale alongside marginally racy reading material, such as English translations of Eugène Sue’s popular novel *Les Mystères de Paris* (1842–43).^29^ Those placed under pseudonyms, such as “Henry Smith,” parallel medical works with more explicit offerings, such as the flagellation fantasy *Venus’s Schoolmis-tress* (ca. 1810), often beneath suggestive headers like “Love’s Doings in Paris” and “Real and Curious French Prints.”^30^ The expanding number of newspaper advertisements listing a combination of erotic literature, nude images, and medical works for sale under new names and addresses in the 1850s highlights the vitality of a commerce in medical eroticism not present thirty years earlier. Works on sexual health had become a staple product of Britain’s emerging pornography industry.

The similarities between commercial medical dealers’ and Holywell Street publishers’ business practices are striking. They advertised similar—sometimes the same—works in many of the same venues, often at comparable prices, and disseminated them using the same methods. Both [Other P-722]groups also used aliases to conceal the infrastructure of their businesses, and often published several advertisements, each under a different name, in a single newspaper issue. For example, in addition to using Robert Jacob Jordan’s own name after he earned medical credentials, the Jordan family firm, one of the “most notorious and time-honoured . . . quack firms” of the period, published, advertised, and practiced under dozens of pseudonyms, mostly derived from the names of eminent medical practitioners.^31^ This practice shielded commercial medical dealers’ businesses as a whole from litigious clients who felt manipulated into paying enormous sums for ineffectual treatments.^32^ At the same time, it stamped the publications that advertised them (and the practice of reading about “secret diseases” and “female problems” itself) with the appearance of elite professional endorsement. Repackaging existing publications was also, as with Holywell Street publishers, a hallmark of these dealers’ businesses: they pirated each other’s works, sold old publications under new titles, and recycled parts of them to create “new” books and pamphlets. For instance, passages from *Manhood: The Causes of Its Premature Decline with Directions for Perfect Restoration*, published by Joseph Lambert under the name John Lewis Curtis in 1842, also appear in the Jordan’s (alias “R. and L Perry and Co.”) *Silent Friend*, first published a year earlier.^33^

However, differences in the ways these groups represented themselves and their wares meant that they initially aroused opposition from separate sectors. Commercial medical dealers catered to readers’ desire for discreet access to expert information on sexual health. Their advertisements for medical publications, using headers like “Manly Health” and “Self-Preservation,” offer more detail about their contents than do those of Holywell Street publishers, and promote their authors as health experts: they highlight their (often spurious) medical qualifications and associations with hospitals and other medical organizations, and provide directions for arranging personal consultations and purchasing proprietary medicines. These dealers’ self-branding as medical authorities enraged other practitioners. By the mid-1840s, a vocal minority had begun to accuse them of peddling obscenity. In contrast, Holywell Street publishers emphasized their medical works’ value as adult entertainment; and, although many sold French letters, they did not represent themselves as medical experts. Dugdale’s ca. 1855 catalogue entry for “Tissot’s Celebrated Work, Onanism Unveiled” makes this explicit: “*There is no medical*[Other P-723]*practice connected with the sale of this work*.”^34^ It was not until the 1850s that these publishers’ medical works became subject to accusations of indecency, this time from groups primarily concerned with suppressing traffic in erotic fiction and images. The next two sections detail early opposition to these entrepreneurs’ activities, showing how it promoted ideas about the nature of “obscene” medical print that anticipated the *Hicklin* test’s formulation of obscenity.

## Obscene Quackery and Contextual Obscenity

Medical journals like the *Lancet* and the *British Medical Journal* are well known for their campaigns on behalf of causes such as improved sanitary conditions in public schools and workhouses. These journals, as well as the *Medical Circular*, represented their campaigns against “obscene quackery” as comparable interventions for the public good. They attacked commercial medical dealers’ advertisements for works on sexual health, personal consultations, and proprietary medicines on the grounds that they offended public decency.^35^ A typical article from the *Lancet* argued that these “obscene quacks” represented a serious threat to public morals, not simply because they circulated “vile trash” on sexual topics, but also because their advertisements for it exposed morally vulnerable readers to allusions to sexuality.^36^ Readers participated in the campaigns, writing to these and other medical journals to denounce commercial medical dealers’ works on sexual health, or to express anxiety at the prospect of putting “an ordinary newspaper into the hands of a female . . . lest they should be shocked by the disgusting advertisements which are emblazoned on its pages.”^37^ Many supported the *British Medical Journal*’s proposal to form a “Society for the Suppression of Fraudulent and Obscene Advertisements,” which would “redeem . . . the periodical press from its present position of hireling servitude to medical swindlers and obscene advertisers.”^38^ These discussions spilled into the newspapers themselves, whose editors publicly attacked each another for publishing indecent advertisements.^39^ This only [Other P-724]magnified the impression campaigning medical journals gave that the routes of public communication were awash with obscenity, subjugated to the machinations of rich “advertising quacks.”

Michael Brown has linked earlier nineteenth-century anti-quackery campaigns with the medical reform movement.^40^ Working within a competitive medical economy, in which practitioners often struggled to make a living, many medical men pushed for legislation that would “unite the scattered members of our profession into one body,” and suppress competition from “irregular” practitioners.^41^ In the absence of such legislation, they used the language of public good to situate practitioners they considered unqualified for medical practice or damaging to the profession’s image outside it, by accusing them of quackery.^42^ As Roy Porter noted, this term does not correspond to any specific practice; rather, its application constitutes a discursive method of controlling professional behavior.^43^ The shift that the *Lancet* and other journals allied with the medical reform movement made in the 1840s—from characterizing quackery primarily as a form of fraud to indicting quacks as rampant purveyors of obscenity—extended this project during a period that witnessed rising public anxiety about the social effects of cheap print.^44^ Although many medical men considered commercial medical dealers’ trade in sexual information a social danger, characterizing this commerce as *obscene* quackery also served their own interests. The language situated works that often functioned as advertising for “irregular” practitioners outside the bounds of the medical profession and public propriety.^45^ At the same time, it linked one goal of medical reform—to stop medical practice under fraudulent credentials—with public concern about cheap print’s effects on morally vulnerable readers. This lent medical reform new currency as an issue of public morals, and reinforced its advocates’ self-branding as protectors of the public interest.

A closer look at early campaigns against obscene quackery shows how they promoted a contextual method of identifying obscenity that would later anchor the *Hicklin* test. Medical men had long urged readers to [Other P-725]scrutinize medical works for subtle signs of quackery. Perhaps because irregular practitioners had been the first to exploit print to attract middle- and working-class patients, many deemed suspect features of a work that betrayed effort to speak to such patients in accessible terms: advertisement through popular periodicals, placards, handbills, or cards; text that included testimonials for its author’s services or directions to access them; titles that used vernacular terms to refer to health; or sale in sealed envelopes. Campaigns against obscene quackery went further. They continuously linked the obscenity of quack medical works with the processes through which they were presented to the public, framing the promotional practices described above both as morally endangering to vulnerable readers and as signs of grossly offensive content within the work itself. Indeed, anti-quackery campaigners focused far less on the content of commercial medical dealers’ works—which, problematically, often conveyed the same information as works by respected medical authors—than they did on their presentation. I have found no evidence that, in doing so, these campaigners were consciously drawing on earlier libel trials in which experts argued that it was a work’s mode of distribution, not merely its content, that made it dangerous to the public.^46^ They turned to this strategy simply because it was expedient. Linking medical works’ decency as reading material and legitimacy as medical science with certain methods of presentation and distribution enabled them to draw a line between orthodox medicine and quackery rooted in *commercial* practice at a time when no clear orthodoxy in *medical* practice existed.

**Figure 2:**
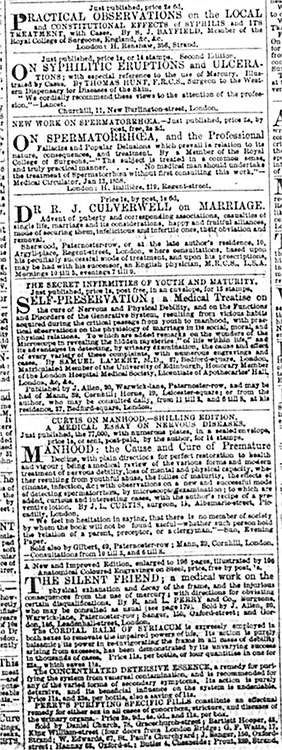
Advertisements for medical publications on sexual topics in *Bell’s Life in London*, February 14, 1858. Print.

In labeling certain works on sexual health obscene, anti-quackery campaigners do not seem to have aroused serious anxiety that medical professionals, as readers of works on such topics themselves, were morally corrupt. Victorian ideologies of reading, which hinged on the notion that male professionals were a special class of reader, one disciplined enough to be unaffected by works that would incite antisocial behavior in others, made this unlikely.^47^ However, campaigns against obscene quackery did risk raising concern that, like commercial medical dealers, “regular” medical authors corrupted vulnerable readers. The dividing line between respectable and supposedly obscene methods of print promotion and distribution [Other P-726]was exceedingly blurry. For example, as Figure 2 shows, publishing firms such as John Churchill, Hippolyte Baillière, Henry Renshaw and Longman, Hurst, Rees, Orme, Brown & Green, which the *Lancet* recommended to would-be medical authors, advertised medical works—including works on sexual health—in some of the same newspapers as commercial medical dealers. These works usually addressed practitioners instead of popular audiences, but they were often distributed through the post at prices affordable for middle-class consumers. Moreover, the newspaper advertisements do not always spell out that these works were written for practitioners, making it even more likely that some so-called vulnerable readers bought them. Campaigning medical journals do not seem to have considered that characterizing public advertisements for works on sexual health as signs of their obscenity, and as obscene themselves, rendered “respectable” medical works vulnerable to the same charges, unless their publishers altered their advertising practices.

I mention this oversight because such changes in the retailing of medical works necessarily followed the introduction of the *Hicklin* test, which relied on a similar model of the line between legitimate and obscene sexual representations. At this stage, though, anti-quackery campaigners led opposition to obscene medical material, and felt very much in control of the narrative about who the “bad guys” were. The possibility of public outcry against “respectable” medical print probably seemed as dim to them as the likelihood of legal interference in its trade. Except in relation medical reform, legislation for which they often represented as a means of eradicating quackery, anti-quackery campaigners rarely depicted the law as a viable means of suppressing obscene medical works. Instead of trying to sue commercial medical dealers for trading in indecent matter, they focused on lobbying the press to stop publishing their advertisements. This proved ineffectual since, as one campaigner acknowledged, these dealers represented a “large and regular” source of revenue that most periodicals did not want to lose.^48^ It was only in the early 1850s, when campaigners began to label public anatomical museums obscene—and their accusations culminated in successful prosecutions of museum owners for displaying “indecent figures” under vagrancy laws—that the law started to become a more important theme in their discussions.^49^ Emerging after commercial medical dealers became associated with such museums as silent partners or owners, and they became outlets for the distribution of works on venereal disease and sexual debility that advertised these dealers’ [Other P-727]

[Other P-728]

consulting services, opposition to public anatomical museums should be understood as an aspect of longer-standing campaigns against obscene quackery.^50^ Accusing such museums of indecency was simply another indirect method of attacking “irregular” medical practice.

The failure of efforts to persuade the press not to publish “quack” advertisements, and the recent application of vagrancy laws to anatomical museums, partly explain why approaches to combating obscene quackery shifted around the passage of the 1857 Obscene Publications Act, Britain’s first piece of legislation against obscenity. Holywell Street publishers’ trade in medical works became subject to debate in this period, when efforts to suppress traffic in erotica resulted in their seizure, beginning in 1856 with the confiscation of “Aristotle’s Works, Physiology of Man, [and] Physiology of Woman” from Edward Duncombe’s premises.^51^ The courtroom debates that followed this and other seizures exposed obscenity law’s potential as a mechanism for suppressing commercial medical dealers’ trade in sexual advice. This possibility altered the nature of discourses about obscene quackery within campaigning medical journals: suppressing trade in “obscene” medical material was increasingly figured as a governmental and judicial responsibility. It also initiated these journals’ participation in a broader public movement that called for stricter policing of the print marketplace.

## Medical Works and the Obscene Publications Act

Courtroom contests over works like *Aristotle’s Masterpiece* situated trade in publications on sexual health as an uncertain legal problem that, for some critics, confirmed the need to resolve disagreement over the definition of obscenity. The term’s meaning had been a sticky legal issue for years, and dogged the passage of the Obscene Publications Act.^52^ As it was originally proposed by the Lord Chief Justice, John Campbell, the legislation enabled the police to seize and destroy obscene materials without a warrant on sworn testimony that they were being sold to the public. The bill garnered immediate criticism because it did not define obscenity, a term that Campbell considered self-evident. Campbell’s aim was to protect [Other P-729]morally vulnerable readers by suppressing commerce in cheap pornography and salacious penny periodicals. His critics argued that, in failing to define the term, he was opening the door for the destruction of “great” art and literature by overzealous police officers, citing publications like Gustave Flaubert’s novel *Madame Bovary* (1856), the object of a recent obscenity trial in France.^53^ Campbell eventually altered the bill so that a magistrate, judge, or justice of the peace had the final say in whether seized works were destroyed. It passed, but this measure did not appease his strongest critics. It was inevitable, the *Law Times* opined, that a few “fanatical” justices would consider works “which lovers of art look upon as art . . . injurious to morals and offensive to decency.”^54^

Historians often claim that no judgments were passed under Campbell’s Act on works whose obscenity was seriously contested until 1868—over a decade after it came into effect.^55^ This assessment overlooks charges brought against several Holywell Street publishers for purveying indecent medical works (among other materials) in 1857 and 1858, following similar charges brought against Dugdale and Duncombe in the year prior to the act’s passage by the Society for the Suppression of Vice (1802–85), a private organization that primarily handled prosecutions for distributing obscenity in this period.^56^ These trials have likely been overlooked because court records were usually censored, and routinely fail to indicate what titles came under scrutiny. Newspaper reports are more forthcoming. Like medical journals, newspapers had their own stakes in debates about obscenity. Many drew large revenues from advertisements for sexually detailed works. Some published stories that anti-vice groups considered indecent; some traded on their reputations as moral guardians; and some exploited anxieties about indecent print to undermine competing papers. With these biases in mind, I have corroborated claims made in these documents by comparing them to court records and all available accounts of the events reported.

Medical works seem to have become an object of these trials serendipitously. In the years bookending the Obscene Publications Act, both the [Other P-730]police and the Society for the Suppression of Vice were trying to eradicate the erotica trade by putting Holywell Street publishers out of business.^57^ However, raids of their premises resulted in the indiscriminate seizure of huge volumes of material, including medical works. These works subsequently became the object of courtroom debates in which, newspaper reports suggest, Holywell Street publishers defended themselves by citing the gray areas between the medical, the artistic, and the erotic. Charged with selling obscene stereoscopic slides in February 1858, for instance, Sydney Powell challenged the court to prove that wares he claimed were “intended for medical men . . . and in no respect more indecent than the exposure of living models in our schools of art” were obscene.^58^ If the court could assure him “*of the point at which the line could be drawn*” between science and obscenity, he swore, “he would pledge himself to observe the law.”^59^ Powell’s appeal to the indistinct borders between science, art, and obscenity echoed earlier arguments made by Dugdale’s daughter and legal adviser on his behalf during a trial that inspired Campbell’s bill, his 1856 countersuit against a representative of the Society for the Suppression of Vice for seizing his publications. Arguing that even a legal seizure would not have been justified, these witnesses emphasized obscenity’s subjective nature, suggesting that only an inordinately prudish reader would consider Dugdale’s publications indecent. Dugdale’s daughter compared his most explicit offerings to nude statues on display in the Crystal Palace, while his adviser so effectively ridiculed the society’s characterization of “Dr. Culverwell’s works” as obscene that he provoked laughter in court.^60^

Most records of Holywell Street publishers’ appeals to the hazy line between science and pornography report an unsuccessful defense. As in Duncombe’s 1856 case, James Thornhill, Edward Morris, Charles Paul, William Wynn, and Henry Blackall each protested when they appeared to summons in the autumn of 1857 that their medical works were “perfectly correct” instructional volumes, only to see them destroyed.^61^ However, there is evidence that judicial authorities disagreed about what constituted [Other P-731]obscenity, and judged medical material unevenly. Wynn’s courtroom discussion of his own confusion about what booksellers “may sell and what [they] may not” suggests that many magistrates considered medical works innocent, so they were never debated in court, or debates about them were never recorded:

[A] work called *The Silent Friend*, which was seized last time, and had been returned as unobjectionable, was now seized again. Then *Aristotle* was condemned, but now they had brought out a new edition, which they thought unobjectionable. In fact, *Curtis on Manhood*, which had been returned, had been transmogrified into *Aristotle*, which had been condemned. (Laughter) The magistrate would, therefore, see all they wanted to do was to “keep within the law.” (Laughter)^62^

The same gray area between the medical and the erotic that allowed publishers like Wynn to market medical works as a marginally legitimate form of sexual entertainment posed a major problem for regulating the print marketplace: there was no commonly agreed upon definition of obscenity to guide dealers in their publishing activities—if they truly wanted to keep within the law—or the authorities charged with policing them.

Reports that record judicial authorities’ comments also show that they had difficulty assessing medical material in the absence of a legal definition of obscenity. In the wake of debates about the Obscene Publications Act’s potential to endanger “great” art and literature, they sought to make judgments in light of the whole work in question, rather than examining isolated passages.^63^ Medical works’ claims to scientific authority suggested, at first glance, that they were not, as a whole, obscene. However, judges and magistrates found it difficult to ignore the fact that these works were sold at low prices to nonprofessional readers alongside material that even Holywell Street publishers often admitted was indecent. As in past cases involving explicit literary works, though, the extent to which the intent of the publisher, how a work was advertised, or to whom it was distributed should function as evidence of obscenity was uncertain.^64^ The magistrate Robert Phillip Tyrwhitt reportedly surmised that “there were certainly some very indecent things” in the medical works that Blackall sold, and that they “were certainly very dangerous to youth,” but, being at least “half-medical,” they must have some scientific value.^65^ Yet perhaps, Tyrwhitt reasoned, “the medical was only used for the purpose of selling [Other P-732]the books.”^66^ After all, the “book before him was never read by young surgeons.”^67^ Ultimately, he condemned Blackall’s medical works, not because he considered them bereft of scientific value, but because Blackall sold them “into the hands of raw, inexperienced youths,” where they “might do them an immense injury.”^68^ Like many anti-quackery campaigners, judicial authorities often situated the question of a medical work’s decency in relation to its audience. For Tyrwhitt, the fact that Blackall’s books were sold to vulnerable readers shifted the nature of their sexual content from necessary scientific detail to gratuitous sexual representation.^69^

The extensive press coverage of these trials suggests significant public interest in their outcomes, coming at the heels of the controversy around the Obscene Publications Act’s passage. That newspapers reported them in different ways also emphasizes that this public was not a uniform mass, but one made up of various groups with different stakes in debates about obscenity. For instance, *Bell’s Life in London* published news on sports, politics, foreign events, entertainment, and crime for a wide male read-ership. It drew substantial advertising revenue from Holywell Street publishers, commercial medical dealers, and respected medical publishers, who all wanted those readers to purchase their works on sexual health. And, although it cannot be classed with the salacious “flash” periodicals that Campbell sought to eradicate, the paper’s own reporting, particularly on crime, sometimes skirted Victorian standards of decency.^70^ These factors suggest why *Bell’s Life*’s accounts of Holywell Street publishers’ trials are sometimes more detailed than others—naming the exact titles under discussion, for instance, where conservative papers like *The Times* often would not—and why they focus more on the defense’s arguments. Parliamentary debates surrounding Campbell’s bill had concentrated on how increased police powers to seize obscene materials could impact wealthy art collectors and the literati. Courtroom debates about medical works showed that the act threatened both *Bell’s Life*’s fortunes and those of its readers, for whom such works represented a rarely (if precariously) permissible source of sexual information and titillation.^71^

If *Bell’s Life*’s editors were concerned that these trials portended more rigorous policing of sexually themed print, their anxieties proved [Other P-733]warranted. Public debate about the print marketplace intensified in the late 1850s and 1860s, and increasingly focused on a problematic category of obscenity, one that encompassed a wide range of works that fell uncertainly between the categories of instruction, artistic expression, and pornography. Campaigning medical journals framed the medical profession as a key stakeholder in this debate. Their appeals to the police and the Society for the Suppression of Vice to charge commercial medical dealers and owners of public anatomical museums with trafficking obscenity under the Obscene Publications Act were not very successful, but they were influential. In order to combat what might be termed “borderline” obscenity, legal experts and cultural critics drew on discourses about obscene quackery to lobby for a legal definition of obscenity that hinged on the modes through which sexually detailed material was presented and distributed.

## Professional Stakes in Obscenity Law and the *Hicklin* Ruling

The medical press unevenly followed the Obscene Publications Act’s passage through Parliament. Many journals did no more than report on the legislation’s progress, and some made no mention of the bill at all. However, those that campaigned against obscene quackery published a lot on the act’s implications for the medical profession. Some writers viewed its passage ahead of legislation for medical reform as evidence of misplaced government priorities. “If we are to have laws . . . for the suppression of obscene publications,” one groused, “why should we not have an Act of Parliament to suppress a traffic [in quack publications] which in its consequences is equally detrimental to the health and happiness of a large portion of the public[?]”^72^ Others saw it as a potential, if problematic, mechanism to combat obscene quackery.^73^ The act did not cover advertisements, so it remained impossible to prosecute commercial medical dealers for advertising sexual advice. But, by enabling the police to seize their works on sexual health and charge them with distributing obscenity, the legislation could be leveraged against them. *Punch*, which exchanged a number of screeds against obscene quackery with the *Lancet*, deemed this “the chief case for Lord Campbell’s Act.”^74^

Even before the act came into effect, therefore, medical men began to press for action against commercial medical dealers and owners of public [Other P-734]anatomical museums under it. The *Lancet* argued that there was “abundant ground to warrant the interference of the Society” for the Suppression of Vice in their activities, and urged the organization to get to it.^75^ “Worrying the Holywell-street vendors is good sport enough,” it coaxed, “but scarcely more successful than lopping of the heads of a Hydra. . . . Surely [obscene quackery is] more deserving of [the society’s] attention, as being calculated to engender that miserable depravity of mind which induces men to purchase the poison vended by traders in obscene publications.”^76^ When, in the following months, the society failed to follow through—even as Holywell Street publishers’ medical works were being found obscene under the act and destroyed—the *Lancet* and its allies stepped up their appeals. They explicitly equated commercial medical dealers with Holywell Street publishers, dubbing them “Holywell Doctors” and “Holywell quacks,” and argued that quackery should be a key focus of the Obscene Publications Act’s application.^77^

This agitation to combat quackery under obscenity law halted briefly with the passage of the 1858 Medical Act, which established a General Medical Council to form and maintain a public register of qualified medical practitioners. For years, anti-quackery campaigners had claimed that medical reform would eradicate quackery, obscene and otherwise. The Medical Act was not designed for this purpose, however, and it swiftly proved ineffectual as a weapon against commercial medical dealers and owners of public anatomical museums. The legislation made it possible to sanction registered practitioners for “conduct unbecoming of the character of a physician”—including indecent advertising—and to prosecute practitioners who falsely claimed to hold formal medical qualifications.^78^ However, many practitioners simply declined to register themselves, leaving them free to advertise as they chose. Unqualified practitioners also continued to represent themselves as “surgeons” and “physicians.” The charge of falsely claiming a medical degree could easily be dodged by purchasing a foreign diploma, or one could simply pay the five-pound fine for committing the crime—a sum not difficult for the most successful dealers to raise.^79^ In this context, opposition to obscene quackery surged to new heights of hyperbole. F. B. Courtenay’s exposé *Revelations of Quacks*[Other P-735]*and Quackery* (1865), based on a series of letters published in the *Medical Circular*, warned parents that if their daughters sent away for commercial medical dealers’ publications, “irreparable moral contamination” would inevitably ensue.^80^ The *Lancet* deemed their books and advertisements acts of “cunning terrorism” against British minds and morals.^81^ These journals attacked public anatomical museums in similar terms, calling for their closure under the Obscene Publications Act.

When, in 1860, local authorities charged William and Louis Lloyd, anatomical museum owners in Leeds, with displaying models “dangerous to public morality” under the act, these efforts seemed at the brink of success.^82^ The Lloyds’ defense that their anatomical models were educational was rejected, and they were destroyed, on the grounds that they were “utterly useless for any scientific object” and “pandered to the worst passions of human nature.”^83^ Prosecutions of small-time commercial medical dealers followed. In 1865, Thomas North, a “hawker,” and Reginald Rudd, “a medicine vendor,” were charged in Bolton Borough Court with distributing obscene books.^84^ As with many Holywell Street dealers’ trials, here the works’ obscenity was framed as a function of their mode of distribution. Rudd’s solicitor argued that “everything recorded in the publications might be met with medical books,” but he agreed with the prosecution that displaying them publicly “had unquestionably a degrading effect.”^85^ Pessimistic about his client’s chances, he asked for a lenient sentence, promising that Rudd would never show his face in Bolton again.^86^ Rudd was fined only twenty shillings, but campaigning medical journals viewed this and the Lloyd case as important precedents that would inspire further action under obscenity law. However, it proved rare. Eight years after the Lloyd case, the *Lancet* was still calling on the police and the Society for the Suppression of Vice to do their duty, and lamenting the law’s weak enforcement.

Two factors largely explain why campaigns against obscene quackery failed to give rise to extensive legal action under the Obscene Publications Act against medical entrepreneurs in the late 1850s and 1860s. One is what one anti-quackery campaigner called “the ‘fine line of demarcation’ . . . between us and them.”^87^ Reportedly, the police and anti-vice societies [Other P-736]were reluctant to charge practitioners with distributing obscenity in the face of uncertainty about who, exactly, counted as a quack, and what, exactly, counted as obscene medical material.^88^ The Society for the Suppression of Vice’s dwindling resources and the fact that efforts to combat the erotica trade—its main concern—were failing probably compounded this hesitancy. The police virtually gave up censuring Holywell Street publishers by the mid-1860s, because fining and even imprisoning them was so ineffective: in their absence, family members simply carried on the business.^89^ Weak support from professional bodies also seems to have hindered direct action. Medical periodicals of all kinds published diatribes against quackery, but, with the exception of the journals I have cited, few published articles and even letters to the editor on *obscene* quackery.^90^ Aside from the *British Medical Journal* (as the organ of the British Medical Association), major medical organizations also expressed little interest in exploiting obscenity law to combat quackery, even though they used accusations of indecency to delimit legitimate practice in other ways: for instance, the General Medical Council refused to admit proprietors of “unseemly” exhibitions into the register, and struck several commercial medical dealers from it for unprofessional conduct.^91^ Only in the 1870s, when doctors associated with the Lock Hospitals and the *Lancet* began to fund private prosecutions through the Society for the Suppression of Vice, did practitioners organize to suppress “irregular” practice under obscenity law.^92^ Even in this case, however, legal action was an independent matter, spearheaded by a group of specialists in venereal disease who most closely competed with commercial medical dealers, and who were among the most threatened by the “fine line of demarcation” that separated them. [Other P-737]

Commercial medical dealers and Holywell Street publishers thus continued a brisk trade in works on sexual matters, prompting the *Saturday Review* to declare the Obscene Publications Act a dead letter in the spring of 1868.^93^ The law, it claimed, would have been difficult to enforce even if the police and anti-vice societies were willing and able to take systematic action: Campbell’s refusal to define obscenity had paved the way for increasing trade in works “which do not exactly fall within the scope of the bill, but which are perhaps better calculated to effect the infamous objects which it attempted to discourage.”^94^ This assessment reflects the character of public discourse about obscenity during the preceding decade, which increasingly focused on “borderline” material. The *Review* and other periodicals that positioned themselves as moral guardians railed at open commerce in works that, in their view, straddled the line between the licit and the illicit, from sensuous poetry, satirical stereoscopic slides, and photographs of ballet dancers to press reports on divorce court proceedings and religious pamphlets.^95^ These organs articulated such works as a problematic category of obscenity, one whose free trade in the streets of the metropolis violated of the spirit of the law. Anti-obscenity legislation’s failure to curb this trade, they argued, necessitated the introduction of a rigorous legal definition of obscenity that accounted for material whose mode of presentation and distribution made it a threat to public morality.

Such critics borrowed from screeds against obscene quackery to authorize claims that “borderline” works were a social danger, and to frame many of these works as calculated “evasions of the law.”^96^ At the same time, legal experts increasingly used medical works as exemplars in their own writings about the need for a legal definition of obscenity to resolve judicial uncertainty about the act’s application. In framing obscenity as a context-dependent condition, both groups repeated arguments that had already been staged in the courts and in campaigning medical journals. Ironically, however, they expressed more concern than medical men did themselves with protecting scientific discourse. When arguing that certain modes of production and dissemination separated legitimate forms of sexually detailed print from criminally obscene works, legal experts suggested [Other P-738]it was necessary to take commercial context into account in obscenity cases precisely because it could *justify* a publication. A sexually detailed medical work written for and disseminated among practitioners could be “technically obscene,” but legitimate, since it was clearly necessary to professional practice.^97^ Press reports on the 1868 *Hicklin* case, which surrounded *The Confessional Unmasked* (1851), a pamphlet that purported to expose the “Depravity of the Romish Priesthood,” also stressed that some “obscene” works were legitimate. Addressing claims that the *Confessional*, which translated into English Latin passages from the works of various Catholic theologians, was simply a religious work, the *Pall Mall Gazette* argued that some “obscene publications are obviously not injurious to the public . . . many medical books . . . contain matter which is grossly obscene . . . but such publications are not criminal, *because the interests of medicine* . . . *require it*.”^98^ “Surely, however, if a man were to pick out every foul passage [from such a work] . . . and to sell them in a penny pamphlet to boys in the streets,” the work should be considered criminal: these actions made it useless to the profession and endangered vulnerable readers.^99^

The test of obscenity that Alexander Cockburn formulated in his ruling on the *Hicklin* case, “whether the tendency of the matter charged . . . is to deprave and corrupt those whose minds are open to such immoral influences and *into whose hands a publication of this sort might fall*,” affirmed the view of obscenity that these critics, and anti-quackery campaigners before them, promoted: it located a publication’s potential to injure public morals not within its content alone, but also within its commercial relation to vulnerable readers.^100^ In creating this test, Cockburn was not drawing solely on these groups’ views. Experts had argued about the bearing that a work’s mode of distribution had on its legal status since at least 1728, the year obscene libel became an indictable offense, in cases surrounding various kinds of works: some literary, some political, some medical.^101^ However, the judge clearly intended that the test resolve the concerns about “borderline” obscenity—and particularly, obscene medical works—that they so publicly and tirelessly expressed. In the larger ruling, he specifically addressed its application to these works, declaring that a “medical treatise may, in a certain sense, be obscene, and yet not the subject for indictment . . . *the needs of the profession require it*.”^102^ Such [Other P-739]a work’s “circumstances of publication” could, therefore, exempt its distributors from prosecution, or it could doom them.^103^ This was a blunt solution to outcry against obscene medical material. Cockburn’s test justified prosecuting dealers that medical and moralist groups deemed a social danger, and made success a virtual certainty. His claim that it also *protected* legitimate medical works from prosecution was more problematic. This claim was based on an assumption about the relationship between print and medicine that threatened to radically contract the distribution of medical knowledge: legitimate medical works were written for, and read by, practitioners alone.

## Conclusion

The *Hicklin* test proved a double-edged sword for the medical profession. As with the Obscene Publications Act, anti-quackery campaigners recognized that it provided a means of suppressing practices that they opposed, and of disciplining professional behavior.^104^ This led to a wave of prosecutions against “obscene quacks” under obscenity law, often litigated by the Society for the Suppression of Vice but funded by medical practitioners. These actions put a number of commercial medical dealers out of business, and newspapers advertisements for such dealers’ works on sexual health did become both less numerous and more confined to down-market venues in the 1870s and 1880s. The number of public anatomical museums in Britain also declined over time.^105^ However, neither disappeared by any means, and direct advertisements for proprietary medicines and consulting services actually became *more* numerous.^106^ Ultimately, it seems, the *Hicklin* test was fairly ineffectual as a weapon against a large and adaptable sexual advice industry.

The test proved more effective for defining what constituted appropriate professional behavior, and for holding practitioners anxious to maintain the support of medical organizations to this code of conduct. Cock-burn’s ruling helped authorize bans against advertising medical works [Other P-740]for professionals in the daily papers, on the grounds that authors who allowed such advertisements to be published were engaging in a covert and unethical form of self-promotion.^107^ It also lent weight to the disciplinary procedures of medical organizations, which moved to strengthen distinctions between legitimate and “indecent” medical behavior in the 1870s and 1880s by censuring practitioners who distributed works on contraception for “infamous conduct in a professional respect.” The General Medical Council often drew from the *Hicklin* ruling’s language in such cases, emphasizing the legal as well as professional risks that this practice now carried.^108^ Although these actions provoked backlash—as with the Freethought activists Annie Besant and Charles Bradlaugh’s republication of Knowlton’s *Fruits of Philosophy* for the purpose of “testing” *Hick-lin*—they dissuaded many progressive practitioners from publishing such works, as organizations like the Malthusian League responded by officially removing their support.^109^ If *Hicklin* both failed to eradicate “quackery” and impeded progressive public health initiatives, it did assist efforts to delineate orthodox medical behavior, and to proscribe stricter codes of conduct for medical communication and publicity.

Importantly, however, the *Hicklin* test was not actually designed to discipline the medical marketplace. Its purpose was to discipline the *print* marketplace. It could, therefore, be applied to works that most medical men considered respectable, without any professional approval. Cockburn’s comments on legitimate “circumstances of publication” were sometimes taken to mean that works on sexual health sold to nonpractitioners but restricted by means of price or distribution from “vulnerable” readers were protected from prosecution.^110^ However, he had not, in fact, specified what these circumstances were. Practically speaking, this meant that the distributors of any medical work to nonpractitioners that discussed sexuality could be prosecuted for selling obscene material, if the right parties were motivated to do so.^111^ Among other works considered legitimate even [Other P-741]by conservative medical men, Cockburn’s vague language famously made possible the indictment of Havelock Ellis and John Addington Symonds’s *Sexual Inversion* (1897) as an obscene book.^112^ Such cases were, however, extraordinarily rare. The spectral risk of prosecution, rather than actual legal action, seems to have impacted “respectable” medical communications much more significantly, making even eminent medical authors hesitant to write on sexual matters. The rising number of disclaimers about the necessity of mentioning sex in medical publications during the last third of the nineteenth century suggests that concerns about prosecution also influenced decisions about composition, editing, and distribution, as authors and publishers felt compelled to anticipate and diffuse objections to their work.^113^

As in the literary and scientific spheres, then, the *Hicklin* test’s most pervasive influence on medical communication was, arguably, extralegal.^114^ Cockburn’s ruling intensified a narrowing of parameters in the nineteenth century through which authors, publishers, and distributors of all types, with all kinds of motives, could disseminate medical knowledge without undertaking the risks of legal and/or professional censure. Having tacitly framed the medical publication’s only legitimate role as a means of communication between professionals, the test impeded communication with the public, hurting the profession as much as it helped it.^115^ Legal entrenchment of this view about print’s purpose for medicine had been a long time coming. Mid-Victorian trade in popular works on sexual health exposed medical print’s generic instability and its complex and sometimes discontinuous relation to medical practice. Although a great deal was said about these works, little serious discussion took place about who *should* publish on medical topics, how medical works *should* be presented, and who *should* read them. Legitimate medical print was usually defined in the negative: talking about what it was not was a tantalizing [Other P-742]tool for furthering interests that lay elsewhere. The *Hicklin* test—which would impact the production, dissemination, and interpretation of so many works across the English-speaking world—inherited oversights from contests over medical print that were, in large part, proxies for battles over control of the markets it was caught between: the medical marketplace and the print marketplace. [Other P-743]

## Footnotes

1
British College of HealthMorality of Modern Medicine MongersLondonBritish College of Healthca. 1852

2
“Mesmeric M.D.,” in ibid.

3
See BrownMichaelMedicine, Quackery, and the Free Market: The ‘War’ Against Morison’s Pills and the Construction of the Medical Profession, c. 1830–c. 1850Medicine and the Market in England and Its Colonies, c. 1450–c. 1850JennerMark SRWallisPatrickBasingstokePalgrave Macmillan2007238261 for further information about Morison and the college’s depiction of competing practitioners.

4
The Queen, on the Prosecution of Henry Scott, *Appellant*, *v*. Benjamin Hicklin and Another, Justices of Wolverhampton, *Respondents*Censorship LandmarksDe GraziaEdwardLondonR.R. Bowker19695119

5
There are many studies that touch on this issue. I have found the following especially informative: BeiselNicolaImperiled Innocents: Anthony Comstock and Family Reproduction in Victorian AmericaPrinceton, N.JPrinceton University Press199711623535 DawsonGowanDarwin, Literature, and Victorian RespectabilityCambridgeCambridge University Press2007116161 HallLeslie ASex, Gender and Social Change in Britain since 18802nd edBasingstokePalgrave Macmillan2013 PuglionesiAlicia‘Your Own Effort Has Been to Create Desire’: Reproducing Knowledge and Evading Censorship in the Nineteenth-Century Subscription PressBull. Hist. Med.89320154634902652166910.1353/bhm.2015.0086 RoyleEdwardRadicals, Secularists and Republicans: Popular Freethought in Britain, 1866–1915ManchesterManchester University Press1980 St John-StevasNormanObscenity and the LawLondonSecker & Warburg1956

6
See AlbertiSamuel JMMMorbid Curiosities: Medical Museums in Nineteenth-Century BritainOxfordOxford University Press2011164196 BatesAW‘Indecent and Demoralising Representations’: Public Anatomy Museums in Mid-Victorian EnglandMed. Hist.52120081221818080910.1017/s0025727300002039PMC2175054 BatesAWDr. Kahn’s Museum: Obscene Anatomy in Victorian LondonJ. Royal Soc. Med.9912200661862410.1258/jrsm.99.12.618PMC167633717139064 ; Maritha Rene Burmeister, “Popular Anatomical Museums in Nineteenth-Century England” (PhD diss., Rutgers University, 2000), 179–239; RosenmanEllen BayukBody Doubles: The Spermatorrhea PanicJ. Hist. Sex.1232003365399 StephensElizabethPathologizing Leaky Male Bodies: Spermatorrhea in Nineteenth-Century British Medicine and Popular Anatomy MuseumsJ. Hist. Sex.17320084214381926361510.1353/sex.0.0023 . Most censorship histories, from St. John-Stevas, *Obscenity* (n. 5) to BradshawDavidPotter’sRachel (eds.) recent Prudes on the Prowl: Fiction and Obscenity in England, 1857 to the Present DayOxford Oxford University Press 2013 ignore pre-*Hicklin* debates about obscene medical material, and do little to explain *Hicklin*’s relationship to prior debates about the nature of print. One exception is McDonald’sPeter DOld Phrases and Great Obscenities: The Strange Afterlife of Two Victorian AnxietiesJ. Victorian Cult.1322008294302 . Although McDonald’s interest is in the test’s relation to Victorian ideas about “great” literature, he acknowledges that the status of medical works was a consideration in its formulation.

7
I draw here on Mary E. Fissell’s discussion of the relationship between medical print and the medical marketplace in “The Marketplace of Print,” in Jenner and Wallis, *Medicine and the Market* (n. 3), 108–32.

8
See KassellLaurenMedical Understandings of the Body, c. 1500–1750The Routledge History of Sex and the Body: 1500 to the PresentToulalanSarahFisherKateLondonRoutledge201357745860 PorterRoySexual Advice before 1800Sexual Knowledge, Sexual Science: The History of Attitudes to SexualityPorterRoyTeichMikulasCambridgeCambridge University Press1994134157 WahlElizabeth SusanInvisible Relations: Representations of Female Intimacy in the Age of EnlightenmentStanford, CalifStanford University Press199930

9
See FyfeAileenSteam Powered Knowledge: William Chambers and the Business of Publishing, 1820–1860ChicagoUniversity of Chicago Press2012 HewittMartin The Dawn of the Cheap Press in Victorian Britain: The End of “Taxes on Knowledge,” 1849–1869London Bloomsbury Academic 2014 for further information about these developments.

10
Compare these dealers’ practices to those described by Fissell in “Marketplace of Print” (n. 7) and PorterRoyHealth for Sale: Quackery in England, 1660–1850ManchesterManchester University Press1989

11
See EliotSimonSome Patterns and Trends in British Book Production, 1800–1919Occasional Papers of the Bibliographical Society819945977 for further information about what constituted “cheap” print in this period. To determine median prices, I analyzed advertisements published in the following newspapers in the years 1842, 1847, 1852, 1857, 1862, and 1867 using Gale Digital Collections’ British Newspapers 1600–1950 and 19th Century UK Periodicals databases: *Aberdeen J*.; *Bell’s Life in London and Sporting Chronicle*; *Berkshire Chronicle*; *Cheshire Observer and General Advertiser*; *Daily Post* (Liverpool); *Derbyshire Times and Chesterfield Herald*; *The Era*; *Freeman’s Journal and Daily Commercial Advertiser*; *Liverpool Mercury*; *John Bull and Britannia*; *Morning Chronicle*; *Preston Guardian*; *Reynolds’s Newspaper*; and *The Standard*. Prices did not vary depending on which papers the works were advertised in.

12
CourtenayFrances BurdettRevelations of Quacks and Quackery: A Series of Letters by “Detector” reprinted from “The Medical Circular”3rd edLondonBaillière, Tindall & Cox186531 , places commercial medical dealers’ collective annual spending on domestic periodical advertisements alone at fifty thousand pounds. Spending estimates made by other anti-quackery campaigners and by commercial medical dealers themselves are similar (see, for instance, The Silent FriendLondonR. & L. Perry1847118 ). Given their origin, they should be viewed with a grain of salt, but they are plausible, given the costs of advertising (even after 1853) and the extent to which these dealers advertised.

13
Burmeister, “Popular Anatomical Museums” (n. 6), 194–202 confirms Rosenman’s speculations in “Body Doubles” (n. 6), 387.

14
For further information about these publishers, their history, and their connection to Holywell Street, see NeadLyndaVictorian Babylon: People, Streets and Images in Nineteenth-Century LondonNew Haven, ConnYale University Press2000149188 SigelLisa ZGoverning Pleasures: Pornography and Social Change in England, 1815–1914New Brunswick, N.JRutgers University Press2002

15
McGrathRobertaSeeing Her Sex: Medical Archives and the Female BodyManchesterManchester University Press20023862

16
McCalmanIainRadical Underworld: Prophets, Revolutionaries, and Pornographers in London, 1795–1840OxfordClarendon1993156

17
MendesPeterClandestine Erotic Fiction in English, 1800–1930: A Bibliographical StudyAldershotScolar Press1993421

18
McCalman, *Radical Underworld* (n. 16), 205.

19
See Mendes, *Clandestine Erotic Fiction* (n. 17), 420–21 and DennisDonnaLicentious Gotham: Erotic Publishing and Its Prosecution in Nineteenth-Century New YorkCambridge, MassHarvard University Press2009133

20
Surviving publications indicate that Holywell Street publishers started dealing in this mixture of works a few years earlier, around 1830. However, since these publishers were well known for falsifying dates, names, and addresses in their publications, I refer here to the date of the earliest advertisements including medical works that I have located. For example, see The New Frisky SongsterBell’s Life in London9 14 1834

21
These works are all listed for sale in “Works Sold by H. Smith,” in DumasAlexandre The Lady of the Camelias [*sic*] London H. Smith [William Dugdale] c.1855 , C.194.a.447(1), British Library.

22
Dugdale’s competitors dealt in virtually the same medical works he did. See catalogues listed in note 27 for further information on their works, prices, and distribution methods. As marginalia in Ward’s New Catalogue of Parisian NoveltiesLondon Wm. Ward ca. 1860 , DA 676, box 8, no. 22, Lilly Library suggests, a few Holywell Street publishers sold medical works at slightly higher prices.

23
For example, see On the Use of Night-Caps, or, Seven Years’ Experience on the Practicability of Limiting the Number of a FamilyLondon J. Turner [William Dugdale] , n.d. 23 24 , PC.30.a.33, British Library.

24
For example, *Flora Fielding; or, The Amours of a Young Lady* in Gems for Gentlemen: A Collection of Amatory Tales and Adventures, no. 1 London William Johns [William John Dugdale] ca. 1850 features a surgeon who makes “a more minute investigation [of Flora’s body] than was necessary . . . instigated by a more powerful motive than a medical one” (4).

25
See the frontispiece to The Secrets of Nature Revealed; or, The Mysteries of Human Procreation and Copulation Considered and ExplainedLondonPrinted for the Booksellers in Town and Country [William Dugdale]1832 , cup.365.a.36, British Library, and “A Case Worth Knowing by All Females,” in *On the Use of Night-Caps* (n. 23), 24–31.

26
Tellingly, Dugdale’s edition of *The Secrets of Nature Revealed* (n. 25) advertises only one other medical publication for sale in its endpapers. The rest of the listings are for erotic fiction and images.

27
This assessment is based on the following catalogues: Books Published and Sold by M. MetfordA Peep into the Holy Land, or, Sinks of London Laid Open!London John Duncombe , n.d. , DA 688.P37, Lilly Library; A Catalogue of Books, Principally Facetious and HumorousThe Exquisite: A Journal of Fun, Frolic, and FacetiaeLondon Edward Dyer , n.d. , DA 676, box 3, no. 30, Lilly Library; A Catalogue of Books, Principally Facetious and HumorousThe Exquisite: A Journal of Fun, Frolic, and FacetiaeLondon H. Smith [William Dugdale] n.d. , DA 676, box 3, no. 31, Lilly Library; Catalogue of Books Published by H. SmithHints to Men about Town, or WaterfordaniaLondon Henry Smith [William Dugdale] n.d. , DA 679.H666, Lilly Library; A Catalogue of Facetious and Humorous BooksLondon Edward Dyer n.d. , DA 676, box 1, no. 51, Lilly Library, USA; Catalogue of Fast and Funny LiteratureLondon H.G. Brook n.d. , DA 676, box 1, no. 2, Lilly Library; Catalogue of a New Assortment of Scarce and Amorous Literature DumasAlexandre The Lady of the Camelias [*sic*] London H. Smith [William Dugdale] n.d. , C.194.a.447(1), British Library; Catalogue of Select Works, Funny, Flash, and FacetiousLondon John Wilson [Edward Duncombe] n.d. , DA 676, box 1, no. 54, Lilly Library; Sold by John WilsonLondon John Wilson [Edward Duncombe] n.d. , DA 676, box 5, no. 22, Lilly Library; *Ward’s New Catalogue of Parisian Novelties* (n. 22); Works Published by H. SmithThe Philosophy of Kissing Anatomically and Physiologically ExplainedLondon Henry Smith [William Dugdale] , n.d., 41132/P, Wellcome Library; and “Works Sold by H. Smith” (n. 21).

28
For examples, see advertisements for *A Sealed Book for Women* and *On Impotence and Sterility* in “Works Published by H. Smith,” 1; for *Aristotle’s Works*, *Geneseology; or the Physiology of Woman*, and *Conjugal Love; or, the Mysteries of Hymen Unveiled* in “Works Sold by H. Smith” (n. 21), 8; and for *Aristotle’s Works* in “Catalogue of a New Assortment,” 1.

29
For example, see Works Publishing by W. DugdaleNorthern Star and Leeds General Advertiser4 13 18442

30
For example, see Just PublishedNorthern Star and National Trades’ Journal12 13 1845

31
Courtenay, *Revelations* (n. 12), 79. See also Burmeister, “Popular Anatomical Museums” (n. 6), 186.

32
Burmeister, “Popular Anatomical Museums” (n. 6), 188–201.

33
Ibid., 184–85.

34
“Works Sold by H. Smith” (n. 21), 5, my emphasis.

35
The organ of the Provincial Medical and Surgical Association (from 1856, the British Medical Association) changed titles several times during the period this article covers. For ease of reading, I refer to it as the *British Medical Journal* in the body of the article, and cite the original title under which individual articles appeared in the notes.

36
Quack AdvertisementsLancet4912191 9 184746

37
The Disgusting Pages of the NewspapersLancet389877 30 1842622623

38
Proposed Society for the Suppression of Fraudulent and Obscene AdvertisementsAssoc. Med. J.1297 22 1853631633

39
For examples of discussions of obscene quackery in the popular press, see Shameful Impurity of the Hull Advertiser’s ColumnsHull Packet and East Riding Times10 27 184318 Remarks on the Present State of the Medical Profession in This Country . . .Leeds Mercury11 2 18447 PublicationsDaily News5 27 18467 Advertisements & Notices: Medical AnnouncementFreeman’s Journal and Daily Commercial Advertiser9 2 18541 ; and Immorality and QuackeryDerby Mercury6 3 18578

40
Brown, “Medicine, Quackery” (n. 3), 238–61.

41
Quoted in ibid., 239.

42
Ibid., 240.

43
Porter, *Health for Sale* (n. 10), vi.

44
See Fyfe, *Steam Powered Knowledge* (n. 9), 101–10, 135–46 for further information about these anxieties.

45
Rosenman, “Body Doubles” (n. 6), 389.

46
See, for example, the case of *R. v. Curll*, examined in SaundersDavidCopyright, Obscenity and Literary HistoryELH5721990431444 , and the case of *Stockdale v. Hansard*, examined in McGrath, *Seeing Her Sex* (n. 15), 38–62.

47
See LeckieBarbaraCulture and Adultery: The Novel, the Newspaper, and the Law, 1857–1914PhiladelphiaUniversity of Pennsylvania Press19993648 for further information about the gendered and classed constructions of “vulnerable” and “capable” readership in this period.

48
Is it Possible to Redeem the Newspaper Press from Its Servitude to Fraud and Obscenity?Assoc. Med. J.1328 12 1853697698

49
Bates, “‘Indecent’” (n. 6), 13.

50
See Bates, “Dr. Kahn’s Museum” (n. 6), 620–21; Burmeister, “Popular Anatomical Museums” (n. 6), 196; Rosenman, “Body Doubles” (n. 6), 387.

51
“Edward Duncombe, of 28 Little Andrew Street, Saint Martins Lane, Charged with Selling Obscene Prints: Defendant’s Statements,” April 16, 1856, Middlesex Sessions of the Peace: Court in Session Collection, MJ/SP/1856/006, London Metropolitan Archives.

52
See ManchesterColin Lord Campbell’s Act: England’s First Obscenity StatuteJ. Legal Hist.9 2 1988 223 241 for further details.

53
Lord Campbell’s BillJurist7 18 1857290

54
Law Times11 14 1857110

55
See, for instance, Manchester, “Lord Campbell’s Act” (n. 52), 234–35 and Katherine Mullin, “Poison More Deadly Than Prussic Acid: Defining Obscenity after the 1857 Obscene Publications Act (1850–1885),” in Bradshaw and Potter, *Prudes on the Prowl* (n. 6), 11–29.

56
RobertsMJDThe Society for the Suppression of Vice and Its Early Critics, 1802–1812Hist. J.2611983159176 details the society’s history. This should be read alongside ManchesterColinObscenity Law and Its Enforcement in the Nineteenth CenturyJ. Legal Hist.2119814561 , which discusses in more detail the society’s activities after the Obscene Publications Act’s passage.

57
Mullin, “Poison More Deadly” (n. 55), 15.

58
Police IntelligenceMorning Chronicle2 18 18588

59
Ibid., my emphasis.

60
Court of the ExchequerReynolds’s Newspaper7 13 185616 . Dugdale’s daughter likely referred to the statues because an 1854 decision to conceal their genitals had been controversial. See PiggottJan RPalace of the People: The Crystal Palace at Sydenham, 1854–1936LondonHurst200452

61
See The Hollywell-Street NuisanceMorning Chronicle10 14 1857 Police Courts: Bow StreetDaily News11 21 1857 Police Intelligence: Bow StreetMorning Post11 21 18577 Seizure of Indecent PublicationsMorning Chronicle11 27 1857

62
Keeping Within the LawBell’s Life in London11 15 18578

63
Mullin, “Poison More Deadly” (n. 55), 15.

64
See n. 46 for examples.

65
See “Seizure of Indecent Publications” (n. 61).

66
Ibid.

67
Ibid.

68
Ibid.

69
Ibid.

70
See Bell’s Life in London and Sporting Chronicle (1822–1886)Dictionary of Nineteenth-Century Journalism in Great Britain and IrelandBrakeLaurelDemoorMarysaGentAcademia Press20094647

71
Leckie, *Culture and Adultery* (n. 47), 62–111, esp. 93–94.

72
It Would Be DifficultLancet7017718 8 1857146

73
For example, see The Spermatorrhoea ImpostureLancet21857537

74
The Chief Case for Lord Campbell’s ActPunch8 22 185773

75
The Action Against Kahn, of Coventry Street, for Extortion: Suppression of Obscene QuackeryLancet7017728 15 1857175

76
Ibid.

77
See Quacks of Advertising ColumnsPunch10 3 1857144 Downing-Street and Holywell-StreetPunch11 7 1857188 Medical AnnotationsLancet70178411 7 1857478479

78
Bates, “Dr. Kahn’s Museum” (n. 6), 621.

79
Ibid.

80
Courtenay, *Revelations* (n. 12), 26.

81
Purification of the PressLancet8521611 28 1865101

82
The WeekBrit. Med. J.s4-11581 7 18601516

83
Ibid.

84
Suppression of QuackeryLancet11 4 1865518

85
Ibid.

86
Ibid.

87
Medical ParasitesLancet7819798 3 1861127

88
Doctors and QuacksSaturday Review7 10 185830

89
Manchester, “Obscenity Law” (n. 56), 52–54.

90
I examined issues of the following periodicals published between 1853 and 1868: *Assoc. Med. J.; Braithwaite’s Retrospect of Med.; Brit. and For. Medico-chirurgical Rev., Brit. Med. J.; Coffin’s Botanical J. and Med. Reformer; Edinburgh Med. J.; Edinburgh Med. and Surg. J.; Glasgow Med. J.; Homeop. Rec.; Hygeist or Med. Reformer and Defender of Liberty of Conscience and Private Judgment; J. of Health and Phrenological Mag.; J. of Psychol. Med.; J. of Public Health & Sanit. Rev.; Lancet; London Med. Rev.; Med. Press and Circ.; Med. Times and Gazette; Proc. of the Roy. Med. & Chirurgical Society of London;* and *Trans. of the Med. Society of King’s College, London*.

91
Lesley A. Hall, “‘The English Have Hot-Water Bottles’: The Morganatic Marriage Between Sexology and Medicine in Britain since William Acton,” in Porter and Teich, *Sexual Knowledge, Sexual Science* (n. 8), 350–66, 351–53. See also Lancet5 7 1864519

92
Bates, “‘Indecent’” (n. 6), 16. See Society for the Suppression of ViceLancet1 4 18533334 Prosecution of the QuacksMed. Press and Circ.11 27 1872468469 Notices to CorrespondentsMed. Press and Circ.1 8 1873 37 for further details.

93

The Streets of London and Public MoralsSaturday Review5 16 1868 646 647 646.

94
Quote from Holywell-Street RevivedSaturday Review8 21 1858180

95
For examples, see Moral SewageSaturday Review12 24 1864776777 M. Dupin on the Social EvilSaturday Review7 8 18654243 Immoral AdvertisementsPall Mall Gazette11 28 1865910 Mr. Swinburne’s DefenceLondon Review11 3 1866482483 London StreetsSaturday Review11 16 1867629630

96
“Holywell-Street Revived” (n. 94), 180.

97
Law of LibelSolicitor’s J. & Reporter2 11 1865302304

98

The Legality of the ‘Confessional Unmasked’Pall Mall Gazette7 13 1867 10 , my emphasis.

99
Ibid.

100
“The Queen” (n. 4), 9, my emphasis.

101
See Saunders, “Copyright, Obscenity” (n. 46), 432–44.

102
“The Queen” (n. 4), 7, my emphasis.

103
Ibid.

104
See Quacks and Abortion: A Critical and Analytical InquiryLancet153 3936 2 4 1899 327 for direct commentary on how the *Hicklin* precedent could be leveraged to combat obscene quackery.

105
Burmeister, “Popular Anatomical Museums” (n. 6), 245.

106
Indeed, Puglionesi, “‘Your Own Effort’” (n. 5), suggests that the decline in newspaper advertisements for commercial medical dealers’ publications may not reflect any decline in their trade: it seems likely that British dealers, like the American entrepreneurs she examines, adapted to the law by developing promotional and distribution methods that made it easier to evade prosecution.

107
For example, see James R. Lane’s 1875 address to the Harveian Society, reprinted in Medical Advertising and Medical ReviewingBrit. Med. J.17331 16 1875727310.1136/bmj.1.733.72PMC229535320747725

108

D’ArcyF The Malthusian League and the Resistance to Birth Control Propaganda in Late Victorian BritainPopul. Stud.31 3 1977 429 448 , quotation on 434. 11630505

109
See ibid., 433 and Dawson, *Darwin* (n. 5), 433.

110
See FisherJoseph RStrahanJAThe Law of the Press: A Digest of the Law Affecting Newspapers in England, India, and the Colonies: With a Chapter on Foreign Press CodesLondonWilliam Clowes & Sons1898242243 for this interpretation.

111
Besant and Bradlaugh pointed out that under the precedent’s terms, even William Benjamin Carpenter’s respected *Principles of Human Physiology* (a work often given out as a prize to schoolchildren) could be destroyed. See Dawson, *Darwin* (n. 5), 124–28 for further details. See also PowellArthurThe Law Specially Affecting Printers, Publishers, and Newspaper ProprietorsLondonStevens & Sons1887183184 , one of several late Victorian legal advice manuals that, in contrast to Fisher and Strahan, *Law of the Press* (n. 110), argues that issuing medical works containing sexual details posed real risks for publishers.

112
Dawson, *Darwin* (n. 5), 157–61.

113
Hall, “‘English Have Hot-Water Bottles,’” 353.

114
See Dawson, *Darwin* (n. 5), 35–36 and Alison Lorna Elizabeth Wee, “Trial and Eros: The British Home Office v. Indecent Publications, 1857–1932” (Ph.D. diss., University of Minnesota, 2003), 77–78, 147 for further details about *Hicklin*’s extralegal effects on scientific and literary print.

115
See, for instance, the discussion about *Hicklin*’s application to the provision of public information on the Contagious Diseases Acts in The Prospect of a PlagueLaw Times8 5 1871248

